# A New Method of Tenotomy

**Published:** 1892-02

**Authors:** 


### A NEW METHOD OF TENOTOMY.

The following are the steps of an operation which was performed in a case of post-hemiplegic contracture of the flexors of the fingers, by W. W. Keen, of Philadelphia. An incision was made, beginning just above the pisiform bone, and extending three inches obliquely upward, its upper end being over the tendon of the flexor carpi radialis. All the flexor tendons having been exposed, each tendon was first split along the middle for an extent of an inch and a quarter, and then, at the two ends of this incision, section of the opposite halves of the tendons was made ; that is to say, the radical half of the vertical slit, and the ulnar half at the other end. The long loose ends of the divided tendon were then made to glide on each other in a vertical direction over a distance of about half an inch, and sewn together by two transverse sutures. The tendon was thus strengthened to the extent of three-quarters of an inch.—*Med. Age. Jour. A. M. Ass.*

